# Eugenics and Public Health

**Published:** 1922-07

**Authors:** Edith Corry


### EUGENICS AND PUBLIC HEALTH.

By EDITH CORRY.

LIFE can offer nothing better than a sound mind and a healthy body, and those who are physically and mentally below the average have a real grievance against society. But in allotting the blame it is important not to draw superficial conclusions, and to ascribe to faulty environment what is really the result of bad heredity. Men are not born equal, and no equality of opportunity will succeed in putting everyone on a level.

We are living in an environment-cure-all age, and it is therefore all-important to examine schemes for social and physical improvement in their eugenic aspect, as well as in the light of their effect upon human character. It must be realised that the most lavish expenditure upon housing, feeding, infant and maternity welfare, etc., will never in itself solve the problem of national degeneracy. According to Sir George Newman, " If we are to grow a sound and healthy race of men, we must begin where all true breeding begins, *at the source*. If we permit ourselves to favour and provide for the unguided propagation of a population of poor physique, or of persons marked from birth with the stigmata of alcohol, venereal disease or mental deficiency, we shall . . . discover that we are building on false foundations, without taking sufficiently into our reckoning the laws of heredity, of transmission and of antenatal infection." [1]

It cannot be denied that in the aggregate the most degraded homes are inhabited by the least desirable people, nor that the birth-rate in this stratum is higher than in any other section of the population, yet so many schemes which are intended to benefit this bad stock appear to take no account of the nature of the elements of which it is composed. A large amount of money and interest is being expended at the present time on infant and maternity welfare, and where this is not of a pauperising nature it is of unquestionable benefit to society. There is, however, a serious danger of the promotion of ill-judged and sentimental legislation, such, for example, as the indiscriminate Endowment of Motherhood. This measure appears to presuppose that motherhood is quite spontaneous and involuntary, and that fatherhood need imply no responsibility of maintenance, both of which are manifestly absurd. A student of human nature will also be inclined to wonder whence are to come the funds for such endowments, if national wealth is reduced to a minimum by the withdrawal of the natural incentives to work.

There is no doubt that Infant Welfare Centres are bringing certain evils more prominently into the public eye. In the report of a Medical Officer of Health the following statement occurs: " We are also confronted with the problem of the actually mentally deficient . . . of the deranged and epileptic and chronically alcoholic and otherwise mentally abnormal mother. The bad mothering of these cases is quite unimprovable at an Infant Welfare Centre, and a very definite, if not relatively very large percentage of our infants are suffering severely as a result of dependence on such mothering."[2] While deploring in general a high rate of infantile mortality, it must be emphasised that if Welfare Centres succeed in checking the high death-rate of the offspring of such mothers, they will be doing a very anti-social piece of work, unless at the same time suitable information on the subject of birth control is allowed to be judiciously given. The familiar couplet

> " Thou shalt not kill, but needst not strive
> Officiously to keep alive "

may be a cynical saying, but it has a foundation of commonsense which is sadly lacking in these days. In popular imagination two well-known fallacies still hold the field, the first being that every baby is born healthy, and the second that every child is a national asset. If the public could be made to grasp the fact that each child born of defective parents may result in a national loss of between £1,000 to £2,000, the question of mental deficiency might be seriously tackled. Not only is the cost of special elementary education of physically and mentally defective children very much greater than that of ordinary schooling, but the cost of institutional treatment now ranges from between £60 to £100 a year.

It is generally known that mental defectives have, as a rule, strong sexual tendencies, and are very prolific, and it is unlikely that birth control will ever really touch this class, yet we should anticipate very strenuous opposition to any proposal to restrict marriage to those of untainted stock. That we have obviously failed in the past to protect the unmarried is evident from some statistical returns of 1920 to which I have access. In one area, 146 women had produced 80 illegitimate children before being dealt with, and 18 had led immoral lives ; in other areas, 176 women on the books of the Association were known to have had 64 children, 98 to have produced 34, 323 had had 134, and 44 of the mothers had led immoral lives. These are a few examples of the morbid fruit which has been produced of recent years. Such children swell the illegitimate birth and death rates, and when one realises that the mothers are often diseased as well as defective, one can only be thankful that a comparatively small proportion of these children survive. There is nothing for it but life segregation in many cases, and how serious a burden this is likely to become may be instanced by a family recently brought to my notice, which was composed as follows :—

(1) Feeble-minded (dead).
(2) Died in infancy.
(3) Feeble-minded and epileptic.
(4) Died in infancy.
(5) Imbecile.
(6) Idiot (dead).
(7) Feeble-minded and epileptic.
(8) Feeble-minded and epileptic.
(9) Backward, too young to diagnose.

If the Mental Deficiency Act had been in force 30 years ago, and the feeble-minded and epileptic mother had been placed in a certified institution, what a saving would have been effected to the rates; for it is obvious that to deal adequately with any appreciable number of similar families might well exhaust the spending powers of any local authority, though the prospects are more hopeful now that the restrictions on expenditure have been removed. One is naturally loth to advocate sterilisation, though it has a few prominent exponents in this country and is in force in several American States; but anyone who has had the experience of noting the annual child born in some of these defective families will be constrained at least to examine the possibilities of this alternative.

Though it is well understood that mental deficiency, epilepsy and lunacy are definitely heritable, public opinion has not yet reached the point of considering parenthood a crime in any persons of seriously tainted stock. That all suggested alterations in the divorce laws should be seriously combated is proof of the need of eugenic teaching in the country. The danger of parenthood in cases of recurrent insanity would almost make one go to the extreme of advocating the immediate annulment of the marriage of anyone found to be certifiably insane. Prohibition of marriage and compulsory divorce would doubtless be considered great hardships, but there is no greater curse than to be born with the seeds of insanity.

Temperance reformers are inclined to include alcohol amongst the racial poisons, but there is no evidence that it has exerted any harmful influence on the offspring when the parents come from sound and healthy stock. On the other hand, experiments on animals indicate that alcohol cannot be altogether ignored as one of the contributory factors in racial decay. All schemes to promote true temperance are of social value, and nothing would do more to improve the general well-being of the nation than the reduction of the drink bill.

The most costly burden that public health work now has to bear is that of venereal disease; but though the symptoms which manifest themselves in the offspring of those afflicted with these diseases are of an appalling nature, syphilis is not actually considered to be a true racial poison. According to Major Darwin, "the best opinion seems to be that congenital syphilitic symptoms are entirely due to the presence of the living microbe in the child during its earlier years; and, if that be so, these falsely called inherited effects give no support whatever to the belief that syphilis may permanently injure the germ plasm, or may produce evil effects for generations to come."[(3)] The question of venereal disease is far too big a one to be tackled here, and in any case is being very widely ventilated at present, and it is to be hoped that measures now being taken to combat it will by degrees have some tangible effect. Public health work in this connection should include very definite instruction and warning to the young, and there is no doubt that more widespread sex teaching should eventually lower the incidence of immorality. Premature and forced marriages are far too often the rule, and the small consideration given altogether to the question of marriage in this country would scandalise any of the savage tribes still existing throughout the world.

The subject of marriage and housing are intimately bound up, and in conclusion a few words must be said on this important question. Though it cannot be denied that bad and insufficient housing is the cause of a great deal of the ill-health and misery in the country, it must be realised that no permanent social regeneration can be expected from a mere improvement in housing. Many of us in the past have watched the transference of unsatisfactory families from hovels to new dwellings, which in their turn have degenerated into slums, for "some birds will foul any nest." In a recent lecture the zoologist of the Scott Expedition related how some of the party, marooned on an island, were able to watch the arrival and settlement of a community of penguins, and how they noticed that in time there developed what might be called a slum area, where the nests were badly made, the birds noticeably quarrelsome and thieving, and even the chicks ill-mannered. Comment is needless!

In Karl Pearson's address on "Eugenics and Public Health" he made a strong point of the danger of drawing sweeping conclusions from insufficient data; for example, in regard to the matter of phthisis and back-to-back dwellings. He maintained[(4)] that it was more true to say that people of poor stock and mentality gravitated towards the worst type of dwelling than that bad housing induced phthisis. The bad qualities found in the parents occupying the back-to-back houses indicated that the Medical Officer of Health who could improve parental habits would achieve twice as much as if he were merely to improve the housing. This is a truism in all health work, but while one admits that good housing will not necessarily be uplifting, one is perfectly aware of the fact that bad housing must inevitably contribute towards the formation of unsatisfactory habits.

To sum up, it must surely be self-evident that no enduring progress is possible unless schemes for social amelioration and racial improvement take into consideration the laws of heredity, and show some real understanding of human nature, and that until reformers and legislators grasp that they have to attack the root and not the fruit, our national health is not likely, in the popular phrase, to get "better and better."

REFERENCES.

1 Sir George Newman: "An Outline of the Practice of Preventive Medicine." Ministry of Health, 1919. Pp. 46 and 47.

2) Dr. Helen Y. Campbell, Bradford.

(3) Major Leonard Darwin: "Preventive Medicine and Eugenics." International Journal of Public Health, Nov.-Dec., 1921, p. 584.

(4) Karl Pearson: "Eugenics and Public Health." London: Dulau, 1912, pp. 29-30.

